# Cetrorelix suppresses the preovulatory LH surge and ovulation induced by ovulation-inducing factor (OIF) present in llama seminal plasma

**DOI:** 10.1186/1477-7827-9-74

**Published:** 2011-05-30

**Authors:** Mauricio E Silva, Juan P Smulders, Monserrat Guerra, Ximena P Valderrama, Claudia Letelier, Gregg P Adams, Marcelo H Ratto

**Affiliations:** 1 Escuela de Medicina Veterinaria, Facultad de Recursos Naturales, Universidad Católica de Temuco, Temuco, Chile; 2 Instituto de Ciencia Animal, Facultad de Ciencias Veterinarias, Universidad Austral de Chile, Valdivia, Chile; 3 Instituto de Anatomía, Histología y Patología, Facultad de Medicina, Universidad Austral de Chile, Valdivia, Chile; 4 Instituto de Producción Animal, Facultad de Ciencias Agrarias, Universidad Austral de Chile, Valdivia, Chile; 5 Department of Veterinary Biomedical Sciences, University of Saskatchewan, Canada

## Abstract

**Background:**

The purpose of the study was to determine if the effect of llama OIF on LH secretion is mediated by stimulation of the hypothalamus or pituitary gland.

**Methods:**

Using a 2-by-2 factorial design to examine the effects of OIF vs GnRH with or without a GnRH antagonist, llamas with a growing ovarian follicle greater than or equal to 8 mm were assigned randomly to four groups (n = 7 per group) and **a) **pre-treated with 1.5 mg of GnRH antagonist (cetrorelix acetate) followed by 1 mg of purified llama OIF, **b) **pre-treated with 1.5 mg of cetrorelix followed by 50 micrograms of GnRH, **c) **pre-treated with a placebo (2 ml of saline) followed by 1 mg of purified llama OIF or **d) **pre-treated with a placebo (2 ml of saline) followed by 50 micrograms of GnRH. Pre-treatment with cetrorelix or saline was given as a single slow intravenous dose 2 hours before intramuscular administration of either GnRH or OIF. Blood samples for LH measurement were taken every 15 minutes from 1.5 hours before to 8 hours after treatment. The ovaries were examined by ultrasonography to detect ovulation and CL formation. Blood samples for progesterone measurement were taken every-other-day from Day 0 (day of treatment) to Day 16.

**Results:**

Ovulation rate was not different (P = 0.89) between placebo+GnRH (86%) and placebo+OIF groups (100%); however, no ovulations were detected in llamas pre-treated with cetrorelix. Plasma LH concentrations surged (P < 0.01) after treatment in both placebo+OIF and placebo+GnRH groups, but not in the cetrorelix groups. Maximum plasma LH concentrations and CL diameter profiles did not differ between the placebo-treated groups, but plasma progesterone concentrations were higher (P < 0.05), on days 6, 8 and 12 after treatment, in the OIF- vs GnRH-treated group.

**Conclusion:**

Cetrorelix (GnRH antagonist) inhibited the preovulatory LH surge induced by OIF in llamas suggesting that LH secretion is modulated by a direct or indirect effect of OIF on GnRH neurons in the hypothalamus.

## Background

In mammals, ovulation implies pulsatile secretion of gonadotropin-releasing hormone (GnRH) from the medio-basal nuclei of the hypothalamus into the hypophyseal portal system, followed by the release of LH from the anterior pituitary into systemic circulation [1]. The classification of mammalian species as either spontaneous or induced ovulators is based on the type of stimulus responsible for eliciting GnRH release from the hypothalamus [2]. In spontaneous ovulators (e.g., cattle, sheep, goats, pigs, horses), release of GnRH from the hypothalamus is triggered when, in the absence of progesterone, systemic estradiol concentrations exceed a certain threshold [3-5]. In induced ovulators (e.g., rabbits, cats, ferrets, camelids), however, neural signals from mating-related stimulation trigger hypothalamic GnRH, followed by the preovulatory release of LH from the pituitary [2]. In spontaneous as well as in induced ovulators, a surge in the circulating concentration of LH is requisite for ovulation.

Authors of early studies [6-9] concluded that alpacas (Lama pacos) and llamas (Lama glama) are induced ovulators based on the observation that ovulation occurred in > 95% of the females subsequent to mounting and penile intromission compared to 14% of the females in which intromission was not allowed. Later studies in llamas [6,10,11] revealed that ovulation is preceded by a sudden rise in plasma LH concentration beginning within 15 minutes of mating.

Recent studies in llamas and alpacas [12-17] have demonstrated the presence of a potent ovulation-inducing factor (OIF) in the seminal plasma of these species, challenging the notion that physical stimulation from copulation is the only mating-related stimulus required to trigger ovulation. In llamas, intramuscular administration of homologous seminal plasma [12-14] and purified llama OIF [15-17] resulted in a rapid increase in plasma LH concentration followed by ovulation [12,13,16,17], similar to that observed after mating [6,10,11]. Biochemical characterization and purification of llama OIF revealed that this factor is a protein with a molecular mass of ≥30 kDa, and is resistant to heat and enzymatic digestion [15]. In a more recent study [16] llama OIF was identified as a 14 kDa protein, suggesting that this molecule may be part of a larger protein complex or be a bioactive form of a larger pro-hormone.

In llamas, the surge in plasma LH concentration triggered by the administration of homologous seminal plasma [12] or purified OIF [16,17] was more sustained than that observed in GnRH-treated females [12]. In this regard, alpaca seminal plasma induced LH secretion in primary cultures of rat pituitary cells [18]. The same effect has been observed in rat pituitary cultures treated with whole seminal plasma [19] or bioactive fractions isolated from the seminal plasma of Bactrian camels [20,21], a related species where semen-induced ovulation has also been described [22]. Moreover, the addition of anti-GnRH antibodies to the rat pituitary cell cultures did not suppress the effect of alpaca seminal plasma on LH secretion [18], supporting the hypothesis that OIF and GnRH are different molecules affecting LH pituitary secretion in a different manner.

Although pervious studies have documented a direct effect of OIF on pituitary gonadotropes in vitro, the role of the hypothalamus in OIF-induced ovulation has not been examined. Gonadotropin-releasing hormone (GnRH) antagonists suppress pituitary gonadotropin secretion by competitive blockade of GnRH receptors [23-25]. Such molecules have been used to study the role of gonadotropins (FSH and LH) in the control of follicular development, ovulation and establishment and maintenance of the CL in several animal species [26-32]. The use of a specific GnRH antagonist that can block the pituitary response to exogenous and endogenous GnRH provides an opportunity to determine, in vivo, whether the OIF-induced preovulatory LH surge is a result of activation of hypothalamic GnRH neurons or a direct effect on the pituitary gland as previously suggested by *in vitro *studies [18-21].

We used an *in vivo *llama model treated with the specific GnRH antagonist, cetrorelix acetate, to determine if the effect of llama OIF on LH secretion is mediated by the stimulation of the hypothalamus or the pituitary gland.

## Methods

### Semen collection and seminal plasma preparation

Semen from 4 adult male llamas was collected twice per week for two months prior to the start of the experiment. Llamas were kept on pasture and were supplemented with hay. They were given water *ad libitum *and were housed indoor at night. Semen was collected with the use of an artificial vagina designed for sheep that was fitted into a phantom mount built of wood and covered with a llama hide [33]. A total of 12 ejaculates were collected from each male llama.

Llama semen was diluted 1:1 (v/v) with phosphate buffered saline (PBS, Gibco, Grand Island, NY, USA) and centrifuged for 30 minutes at 1500 × g at room temperature. The supernatant was decanted to remove spermatozoa and a drop was evaluated by microscopy to confirm the absence of cells. If spermatozoa were observed, the sample was centrifuged again in a like manner. Sperm-free seminal plasma was stored at -80°C. Upon thawing, the diluted seminal plasma was pooled and sonicated to reduce viscosity, as previously described [16]. After sonication, seminal plasma was centrifuged at 10,000 × g for 20 minutes to remove particulate matter.

### Llama OIF purification

Purification of OIF was performed in a 2-step procedure, as previously described [16]. In brief, llama seminal plasma was loaded into a Type 1 macro-prep ceramic hydroxylapatite column (1 cm × 10 cm, 20 μm, BIO-RAD laboratories, Hercules, CA, USA) previously equilibrated with 10 mM sodium phosphate at a pH of 6.8. Elution was carried out at room temperature using a lineal gradient with 350 mM sodium phosphate, pH 6.8, and a flow rate of 0.5 ml/min. An eluted fraction showing a major protein on SDS-PAGE, was concentrated in phosphate buffered saline (PBS, pH 7.4) using a 5 kDA cut- off membrane filter device (Vivaspin, Sartorius, Goettingen, Germany) and subsequently loaded onto a gel filtration column (SEC, Hi Prep™ 26/60 Sepahacryl™ S-100, Amersham Laboratories, Piscataway, NJ, USA). The purification procedure was carried out at room temperature at a flow rate of 0.5 ml per minute using fast protein liquid chromatography (FPLC, Amersham Laboratories). Elution was performed isocratically using PBS at pH 7.4. The bioactive fraction after gel filtration was identified using an *in vivo *llama ovulation bioassay [16] and it was defined as purified llama OIF.

### Experimental design

Non-pregnant, non-lactating female llamas, 4 to 8 years of age, and weighing 135 to 150 kg were examined daily by transrectal ultrasonography using a 7.5 MHz linear-array transducer (Aloka, SSD-500, International Clinics, Chile). Using a 2-by-2 factorial design to examine the effects of OIF vs GnRH with or without a GnRH antagonist, llamas with a growing ovarian follicle ≥8 mm were assigned randomly to four groups (n = 7 per group) and **a) **pre-treated with 1.5 mg of GnRH antagonist (cetrorelix acetate, Cetrotide, Serono, Chile) followed by 1 mg of purified llama OIF, **b) **pre-treated with 1.5 mg of cetrorelix followed by 50 μg of GnRH (gonadorelin acetate, Ovalyse, Pfizer, Santiago, Chile), **c) **pre-treated with a placebo (2 ml of saline) followed by 1 mg of purified llama OIF or **d) **pre-treated with a placebo (2 ml of saline) followed by 50 μg of GnRH. Pre-treatment with cetrorelix or saline was given as a single slow intravenous dose 2 hours before intramuscular administration of either GnRH or OIF. The dose of cetrorelix (10 μg/Kg of body weight) was based on previous reports of effective suppression of pituitary LH secretion in several animal species [27,31,32]. Treatment with GnRH or OIF was given 2 hours after cetrorelix or placebo administration, based on a previous report that demonstrated rapid blockade of LH secretion after intravenous administration of the GnRH antagonist [34].

The ovaries were examined by transrectal ultrasonography every 12 hours from the day of treatment (day 0) to day 2 to detect ovulation. Ultrasonographic examination was performed every-other-day thereafter until day 16 to monitor CL growth and regression. Ovulation was defined as the sudden disappearance of a follicle ≥8 mm detected during the previous examination.

Blood samples for measurement of plasma LH concentration were collected in heparinized tubes every 15 minutes from 1.5 hours before to 8 hours after GnRH- or OIF-treatment. A jugular catheter (o.d. 1.5 mm; i.d. 1.0 mm) was fixed in place one day before sampling to minimize the effects of handling stress on plasma LH concentration. After collection, blood samples were centrifuged at 1500 × g for 10 min and the plasma was stored at -20°C. Plasma LH concentration was determined by a double-antibody radioimmunoassay using ovine radio-iodinated LH (LER 1374-A), ovine antiserum CSU-204 and ovine LH standard oLH-S25 (provided by NIADDK, USA) in 200 μL duplicates, as previously described [35]. Intra and inter-assay CV were 5 and 7%, respectively. The minimal detectable LH dose, defined as 90% of buffer control, was 0.1 ng/mL. The LH assay used in this study was validated previously for LH determination in Pudú (Pudu puda) and alpacas (Lama pacos) [36,37]. For this study, serial dilution of llama plasma with high LH concentration was assayed in the ovine RIA and was plotted against the standard curve. Percentage of binding for each dilution was paralleled to the standard curve.

Blood samples were collected into heparinized tubes (Vacutainer Systems, Becton Dickinson, Franklin Lakes, NJ, USA) by jugular venipuncture every-other-day from day 0 to day 16 to determine plasma progesterone concentration. Blood samples were centrifuged at 1500 × g for 10 minutes and plasma was stored at -20°C. Plasma progesterone concentration was determined using a commercial solid-phase radio-immunoassay kit (Coat-a-Count total progesterone, DPC; Diagnostic Products Corporation, Los Angeles, CA, USA) as previously reported [12]. The intra-assay coefficients of variation were 4.5%, 2.3% and 2.6% for the low, medium and high-reference plasma (means: 1.8, 3.5, and 16.5 ng/mL). The inter-assay coefficients of variation for low, medium and high-reference plasma were 7.0%, 5.0% and 6.0%, respectively.

The study was conducted during August to September at the Universidad Austral de Chile, Valdivia, Chile (39° 38'S - 73° 5'W and 19 m above sea level). All procedures were performed in accordance with the animal care protocols established by the Universidad Austral de Chile and were revised and approved by its bioethics committee.

### Statistical analyses

Statistical analyses were performed using the Statistical Analysis System software package SAS Learning Edition, version 4.1 (SAS Institute Inc., Cary, NC, USA, 2006). Serial data (plasma LH and progesterone concentration profiles, and CL diameter) were analyzed as a 2-by-2 factorial design for repeated measures using the MIXED procedure. The analysis included main effects of antagonist (cetrolix vs placebo), treatment (GnRH vs OIF), time, and their interactions. If significant (P ≤ 0.05) main effects or interactions were detected, Tukey's post-hoc test for multiple comparisons was used to locate differences. Non-serial data (i.e. follicle diameter at the time of treatment, first day CL detected, CL diameter on day 8, maximum CL diameter, day of maximum CL diameter and day of onset of luteal regression) were compared using analyses of variance. Ovulation rates were compared among groups by chi-square analysis. All values are expressed as mean ± SEM.

## Results

The diameter of the largest follicle at the time of treatment did not differ among groups (P = 0.85, Table 1). No ovulations were detected in llamas pre-treated with cetrorelix, irrespective of subsequent treatment with GnRH or llama OIF. The ovulation rate did not differ (P = 0.89) between placebo+GnRH (6/7, 86%) and placebo+OIF groups (7/7, 100%; Table 1).

**Table 1:** Effect (mean ± SEM) of pre-treatment with cetrorelix on ovulation and CL development in llamas treated with GnRH or purified llama OIF (day 0 = day of treatment)

	cetrorelix		placebo	
	GnRH	Llama OIF	GnRH	Llama OIF
	(n = 7)	(n = 7)	(n = 7)	(n = 7)
Follicle diameter at treatment (mm)	11.4 ± 0.4	11.2 ± 0.7	10.7 ± 0.4	10.9 ± 0.5
Ovulation rate (%)	0/7^a ^(0%)	0/7^a ^(0%)	6/7^b ^(86%)	7/7^b ^(100%)
1^st ^day CL detected *	-------	-------	2.3 ± 0.3	2.4 ± 0.4
CL diameter at day 8 (mm) *	-------	-------	13.7 ± 0.5	13.2 ± 0.4
Maximum CL diameter *	-------	-------	13.7 ± 0.5	13.2 ± 0.4
Day of maximum CL diameter *	-------	-------	8.3 ± 0.6	8.4 ± 0.4
Day of onset of CL regression *	-------	-------	10.3 ± 0.6	10.4 ± 0.4

a, b Proportions with different superscripts are different (P < 0.001)

* CL data was collected in 6 and 7 ovulated females in the placebo+GnRH and placebo+OIF treated group respectively.

**Figure 1:**
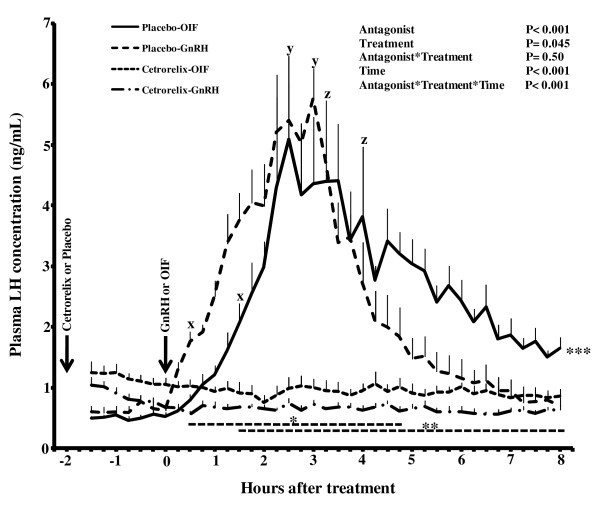
**Effect of cetrorelix pre-treatment on plasma LH concentration in llamas treated with GnRH or llama OIF**. **^x ^**Within group, the first significant increase from pretreatment concentration (P < 0.01). **^y ^**Within group, the maximum concentration (P < 0.01). **^z ^**Within group, the first significant decrease from maximum concentration (P < 0.01). * Interval during which values in placebo+GnRH treated group were higher (P < 0.01) than in both antagonist treated groups. ** Interval during which values in placebo+OIF treated group were higher (P < 0.01) than in both antagonist treated groups. *** Within group, the last value is higher than the pre-treatment value (P < 0.01).

During the 8-hour period after GnRH or OIF treatment, plasma LH concentration increased and decreased (P < 0.01) in both placebo-treated groups, but no change in plasma LH concentration was detected in cetrorelix-treated llamas (Figure 1). The first significant increase in plasma LH concentration was detected 30 and 90 minutes after treatment in placebo+GnRH and placebo+OIF groups, respectively (Figure 1). Maximum plasma LH concentration did not differ (P = 0.16) between the two placebo-treated groups and occurred 2.5 and 3 hours after treatment with OIF (5.1 ± 1.4 ng/mL) or GnRH (5.8 ± 0.5 ng/mL), respectively (Figure 1). In the placebo groups, plasma LH concentrations began to decrease (P < 0.01) at 3 hours and 15 minutes after treatment with GnRH and at 4 hours and 45 minutes after treatment with OIF (Figure 1). Plasma LH concentration decreased to basal levels by 5 hours after treatment with GnRH, but had not yet declined to pretreatment levels (P < 0.01) by the end of the 8-hour post-treatment sampling period in those treated with OIF (Figure 1).

**Figure 2:**
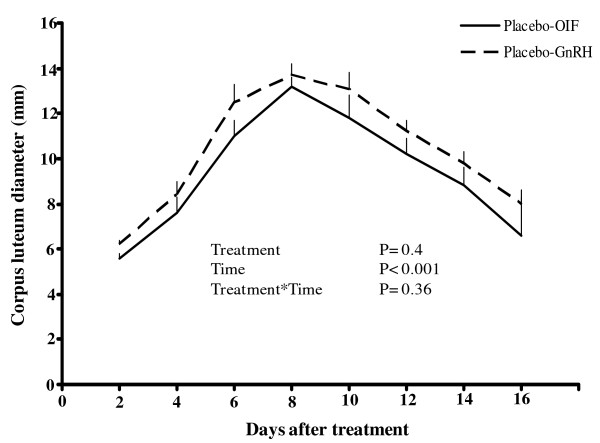
**Corpus luteum diameter in llamas given a placebo pre-treatment and treated with GnRH or llama OIF**.

**Figure 3:**
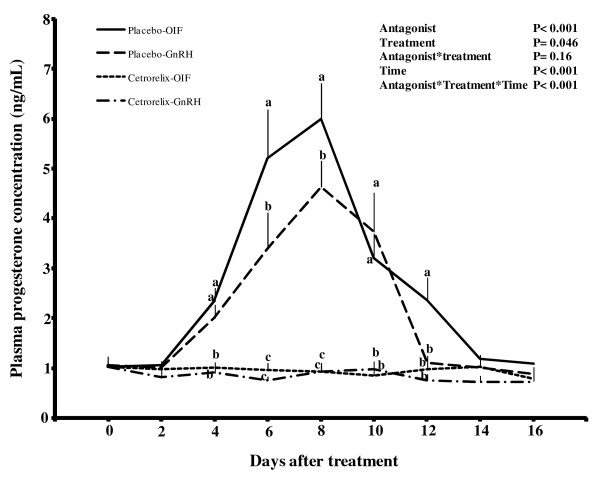
**Effect of pre-treatment with cetrorelix on plasma progesterone concentration in llamas treated with GnRH or llama OIF**. ^abc ^On a given day, values with no common superscript are different (P < 0.01).

The diameter profile of the CL did not differ (P = 0.4) between the two placebo-treated groups (Figure 2). No differences were detected between the GnRH- and OIF-treated groups regarding the first day of CL detection, CL diameter, or the day of onset of CL regression (Table 1). Plasma progesterone concentrations were higher (P < 0.001) in both placebo-treated groups compared with cetrorelix-treated groups where progesterone concentration remained basal throughout the sampling period (Figure 3). In both placebo-treated groups, plasma progesterone concentration increased sharply to peak values by day 8 after OIF (6.0 ± 0.7 ng/mL) and GnRH (4.6 ± 0.5 ng/mL) treatment, and decreased sharply afterwards to nadir by day 12 and 14, respectively (Figure 3). Plasma progesterone concentration was higher (P < 0.05) in the OIF- than the GnRH- treated group on day 6, 8 and 12 after treatment.

## Discussion

The results of the present study provide compelling evidence that llama OIF stimulates LH pituitary release by a previous action on the GnRH hypothalamic neurons. The lack of stimulatory effect of OIF on LH secretion after blocking GnRH receptors on gonadotropes supports the concept that the hypothalamus is the primary target for the action of OIF *in vivo*. Therefore, it is valid to suggest that pituitary LH secretion induced by OIF is triggered by stimulation of hypothalamic GnRH release within the hypophyseal portal vasculature. The administration of cetrorelix acetate (1.5 mg; 10 μg/Kg) as a single intravenous bolus 2 hours before treatment effectively blocked pituitary GnRH receptors and suppressed LH secretion, thereby preventing ovulation normally induced by the administration of a standard dose of GnRH used in this species [38]. Intravenous administration of cetrorelix in the present study blocked GnRH receptors in gonadotropes within 2 hours, similar to the effect described previously [34]. The immediacy of effect allowed us to challenge the females with ovulation-inducing treatments (GnRH or OIF) shortly after antagonist administration, thus avoiding possible confounding effects of longer exposure (e.g., loss of ovulatory capability of dominant follicles).

Based on *in vitro *studies in which alpaca seminal plasma [18], Bactrian camel seminal plasma [19], and its bioactive fractions [20,21] are able to induce LH secretion from rat pituitary cultures, we expected that purified OIF would trigger an LH surge in llamas pre-treated with GnRH antagonist. However, cetrorelix blocked the LH surge as effectively in the OIF-treated group as it did in the GnRH-treated group, demonstrating that both molecules require functional GnRH receptors at the gonadotrope to induce a rise in plasma LH concentration. The inference from these results is that purified OIF induces pituitary LH secretion *in vivo *by stimulation of GnRH release from the hypothalamus. The route by which OIF reaches the hypothalamus, however, is not clear: OIF it is a 14 kDa protein molecule [16] that presumably would not easily cross the blood-brain barrier. However, authors of recent report [39] suggest that peptides of peripheral origin can reach neuronal axons at the median eminence and elicit a direct or indirect effect on the hypothalamus.

The *in vitro *effect of alpaca or Bactrian camel seminal plasma on LH secretion from pituitary cells and the presence of GnRH immuno-reactivity in human seminal plasma [40,41] supported the hypothesis that OIF could be related to the GnRH peptide. However, this has been ruled out by observations that OIF induces a different *in vivo *LH secretion pattern compared to GnRH [12], and the addition of anti-GnRH antibodies to *in vitro *rat pituitary cell culture did not block the LH-releasing effect of alpaca seminal plasma [18]. Moreover, recent biochemical characterization [15,16] has revealed that the molecular mass of llama OIF is much greater than that of the decapeptide GnRH.

Although the hypothalamus is a primary target for the action of llama OIF, the action at the level of the pituitary gland remains unclear. Inconsistencies between our results and *in vitro *effects of OIF [18-21] require further investigation. A plausible explanation may be related to the OIF concentration used in the *in vitro *studies. Purified OIF represents approximately 60% of the total protein in a llama ejaculate (i.e., OIF concentration of 3 mg/ml of ejaculate) [16]; therefore, primary pituitary cultures stimulated with whole seminal plasma [18,19] were exposed to very a high concentrations of OIF in the media. Furthermore, seminal plasma is a complex fluid where cytokines, among other components [42,43], may influence gonadotrope LH secretion [44,45].

Similar discrepancies in LH secretion have been reported for kisspeptin, a key signaling molecule in the neuroendocrine regulation of gonadotropin secretion [46-48]. Kisspeptin effectively induces LH secretion from rat pituitary cell primary cultures [49,50], but when sheep were passively immunized with anti-GnRH antibodies, Kisspeptin did not elicit LH secretion from the pituitary gland [51]. Differential effects observed between *in vitro *and *in vivo *studies of kisspeptin have been attributed to different dosages used [51].

In the present study, intramuscular administration of purified OIF induced a surge release of LH resulting in a high rate of ovulation in placebo pre-treated llamas. A similar effect was demonstrated in previous studies in which llamas were given homologous seminal plasma [12-14] or purified OIF [15-17]. In the present study, plasma LH concentration began to increase within 30 minutes of OIF treatment, peaked at 2.5 hours and declined thereafter, similar to the pattern described in response to mating [6,10,11], or intramuscular administration of whole seminal plasma [12] or purified OIF [16,17]. However, in the present study as well as in a previous one (12), both the rise and fall in plasma LH concentration were delayed in llamas treated with OIF compared to those treated with GnRH, supporting the notion that OIF and GnRH are different molecules. In particular, LH release was sustained beyond the 8-hour sampling period in OIF-treated animals whereas LH concentration was basal by 6 hours after GnRH treatment.

Previous studies support the concept that the ovulation-inducing effect of llama and alpaca seminal plasma is meditated via a systemic rather than a local route, based on the differential ovulation rates obtained after administration of seminal plasma by intramuscular [12,13] and intrauterine routes [13]. While the design of the earlier studies [13] did not rule out potential local effects, results of the present study provide unequivocal confirmation of a systemic route of action since blockade of GnRH receptors at the gonadotrope completely abolished the LH surge and ovulation. This concept is further reinforced by the observation that llama seminal plasma induces a plasma LH surge in ovariectomized llamas [52].

The luteotrophic effect of OIF treatment reported in previous studies (12, 17) was confirmed in the present study. Although the CL diameter profile did not differ between OIF- and GnRH-treated llamas, plasma progesterone was elevated in the former. The relationship between the degree of luteogenesis and the duration of the preovulatory LH surge elicited by OIF warrants further investigation, but is consistent with studies in primates and laboratory species [53-56].

Based on the observation that the GnRH antagonist, cetrorelix, blocked the preovulatory LH surge induced by OIF in llamas, we conclude that the effect of OIF is mediated at the level of the hypothalamic GnRH neurons.

## Competing interests

The authors declare that they have no competing interests.

## Authors' contributions

MS participated in designing the study, acquisition, analysis and interpretation of data, and in writing and revising the manuscript. JPS, MG, XV participated in the acquisition and interpretation of the data. CL and GPA participated in analysis and interpretation of data, and critical revision of the manuscript. As Principal Investigator, MR participated in the intellectual and experimental design of the study, the acquisition, analysis and interpretation of data, as well as writing and revising the manuscript. All authors read and approved the final manuscript.
